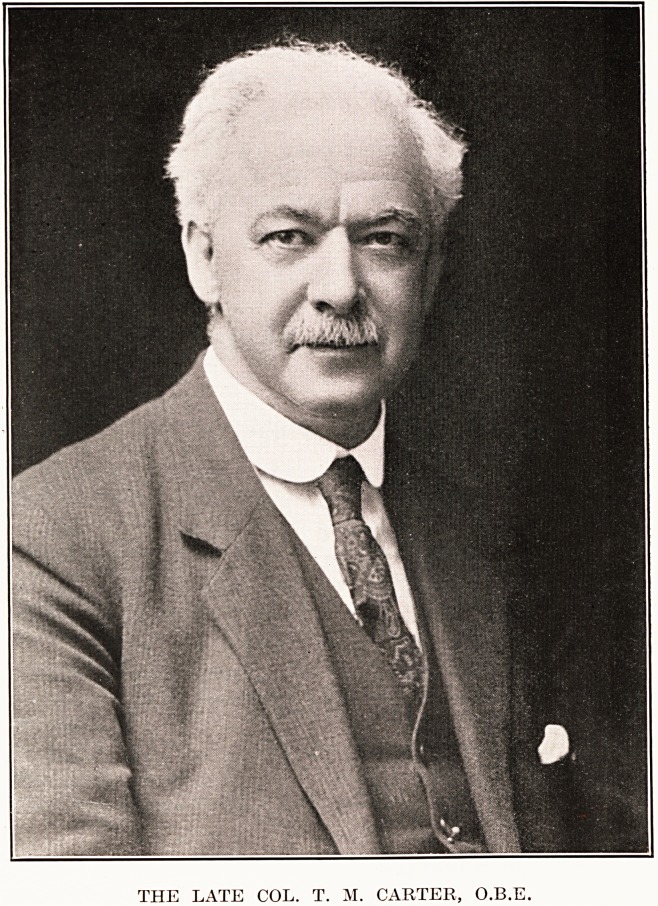# Thomas Moravian Carter

**Published:** 1930

**Authors:** 


### Obituary.

---

### THOMAS MORAVIAN CARTER, O.B.E., M.D.

It is with great regret that we record the death on the 21st February, 1930, at his residence, 19 Westfield Park, Clifton, of T. M. Carter, O.B.E., T.D., M.D., in his sixty-second year.

Dr. Carter was educated at the Bristol Grammar School and University College, Bristol, where he won the Committees' Gold Medal in 1891. He qualified as M.R.C.S., L.R.C.P. in the same year, and held the post of House Physician, and later House Surgeon, at the Bristol General Hospital. He subsequently took up practice in Bristol, and continued here until the outbreak of war in 1914. During part of that time (1904–1910) he was a member of the City Council. He served for many years in the Volunteer and Territorial Forces, and in 1914 was Senior Major of the 6th Gloucesters. When the 2nd Line Battalion was formed he was given command of it, with the rank of Lieut.-Colonel, and proceeded to France in 1916 with the 61st Division. Later on in 1916 he transferred to the R.A.M.C., and saw service in Egypt and the Mediterranean. Before the end of the war he returned to France, and at the Armistice was in command of No. 3 Convalescent Depôt at Boulogne. His success in this capacity made No. 3 Convalescent Depôt one of the pattern depôts, and for this service he was made O.B.E. For some years he was Chairman of the Clifton Industrial School.

Dr. Carter had been West of England representative on the National Health Insurance Committee before the war, and in 1920 was appointed a regional Medical Officer in the Midlands. He returned to Bristol in 1925, to take up a similar appointment here for the South-Western District, and this he held at the time of his death.

Dr. Carter was keenly interested in Freemasonry, and had been Master of the St. Vincent (1404) and Robert Thorne (3663) Lodges in Bristol. He was also an active member of the Quatuor Coronati Lodge (2076).

THE LATE COL. T. M. CARTER, O.B.E.

He was a member of the British Medical Association, Bristol Medico-Chirurgical Society, and the Bristol Shakespeare Society, and was Hon. Secretary of the Bristol and Gloucestershire Archæological Society.

Dr. Carter married in March, 1895, Ada Clara, youngest daughter of the late Mr. H. W. Just, Master of Modern Languages at the Bristol Grammar School, and leaves a son, Major E. T. Graham Carter, R.E., and three daughters.

We offer our deepest sympathy to his widow and children.

### Meetings of Societies.

### Bristol Medico-Chirurgical Society.

## Figures and Tables

**Figure f1:**